# Supplementary material for: BUB1 Inhibition Overcomes Radio- and Chemoradiation Resistance in Lung Cancer
Source: Cancers (Basel). 2024 Sep 27;16(19):3291. doi: 10.3390/cancers16193291 (PMC11475950; doi:10.3390/cancers16193291)
Supplement: Supplementary file 1 [file cancers-16-03291-s001.zip › cancers-3181850-supplementary.pdf]

Supplementary Table S1. Gene mutations in lung cancer cell lines.

Gene Mutation	*A549*	*H2030*	*H1975*	*Calu-1*	*NCI-H2198*	*NCI-H1876*
**KRAS**	Mutant (p.G12S)	Mutant (p.G12C)	WT	Mutant (p.G12C)	-	-
**TP53**	WT	Mutant (p.G262V)	Mutant (p.R273H)	Mutant (gene deletion)	Mutant (p.V157F)	Mutant (p.R273L)
**EGFR**	WT	WT	Mutant (p.T790M)	WT	-	-
**RB1**					-	Mutant

Supplementary Table S2. Primer sequences used in quantitative PCR (qPCR).

Target Gene	***Primer***	***Sequence (5'-3')***	***Tm⁰***
GAPDH	Forward	TCGGAGTCAACGGATTTG	62.4
	Reverse	CAACAATATCCACTTTACCAGAG	59.1
BAX	Forward	TCTGAGCAGATCATGAAGAC	58
	Reverse	TCCATGTTACTGTCCAGTTC	57.5
BCL2	Forward	GATTGTGGCCTTCTTTGAG	59.8
	Reverse	GTTCCACAAAGGCATCC	59
PCNA	Forward	CTGTGTAGTAAAGATGCCTTC	55.6
	Reverse	TCTCTATGGTAACAGCTTCC	56
CASP3	Forward	AAAGCACTGGAATGACATC	57.6
	Reverse	CGCATCAATTCCACAATTTC	62.8
CASP9	Forward	CTCTACTTTCCCAGGTTTTG	57.9
	Reverse	TTTCACCGAAACAGCATTAG	60.3
TP53BP1	Forward	AAGATACTGCCTCATCACAG	56.8
	Reverse	GTATTAGCATCCACATCAGAC	56.1

Supplementary Table S3. Half-maximal inhibitory concentration (IC_{50}) of chemotherapy drugs in lung cancer cell lines.

Cell lines	***BUB1i (μM)***	***cisplatin (μM)***	***paclitaxel (nM)***	***olaparib (μM)***
**A549**	1.1	4	3.3	2.4
**NCI-H2030**	1.6	9.5	18.2	48.7
**NCI-H1975**	1.9	5.8	5.8	16
**Calu-1**	2.8	4.8	13.2	22.1
**NCI-H2198**	5.1	>30	6	17
**NCI-H1876**	4.2	44	2.5	3.1
**PCS-300-010**	15.9	35	11.5	41

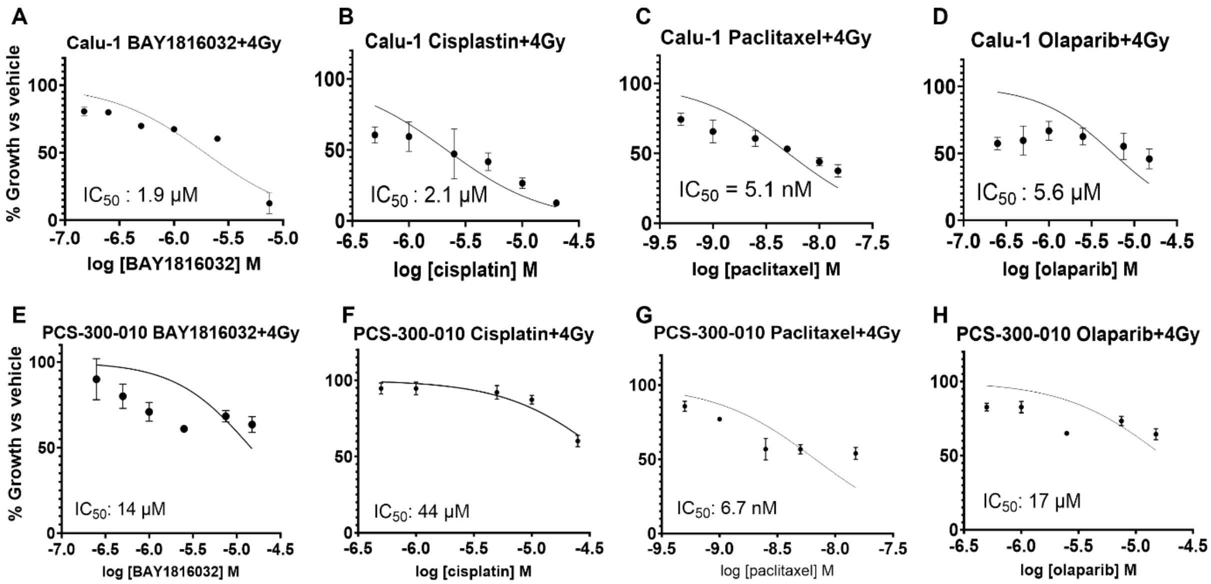

Supplementary Figure S1: The effect of radiotherapy on single agent IC50 of BUB1i, cisplatin, paclitaxel and olaparib in Calu-1 and PCS-300-010 cells. (A–D), Radiation (4 Gy) enhanced cell killing (i.e., reduced IC50) when combined with BUB1i, cisplatin, paclitaxel and olaparib in Calu-

1 cells. (E–H), there was insignificant effect of IR on single agent BUB1i and cisplatin in PCS-300-010 bronchial epithelial cells while the IC50 of paclitaxel and olaparib reduced with radiotherapy in these cells.
