# Supplementary figures and images for: BUB1 Inhibition Overcomes Radio- and Chemoradiation Resistance in Lung Cancer
Source: Cancers (Basel). 2024 Sep 27;16(19):3291. doi: 10.3390/cancers16193291 (PMC11475950; doi:10.3390/cancers16193291)

**File S1.** The original Western blot figures.

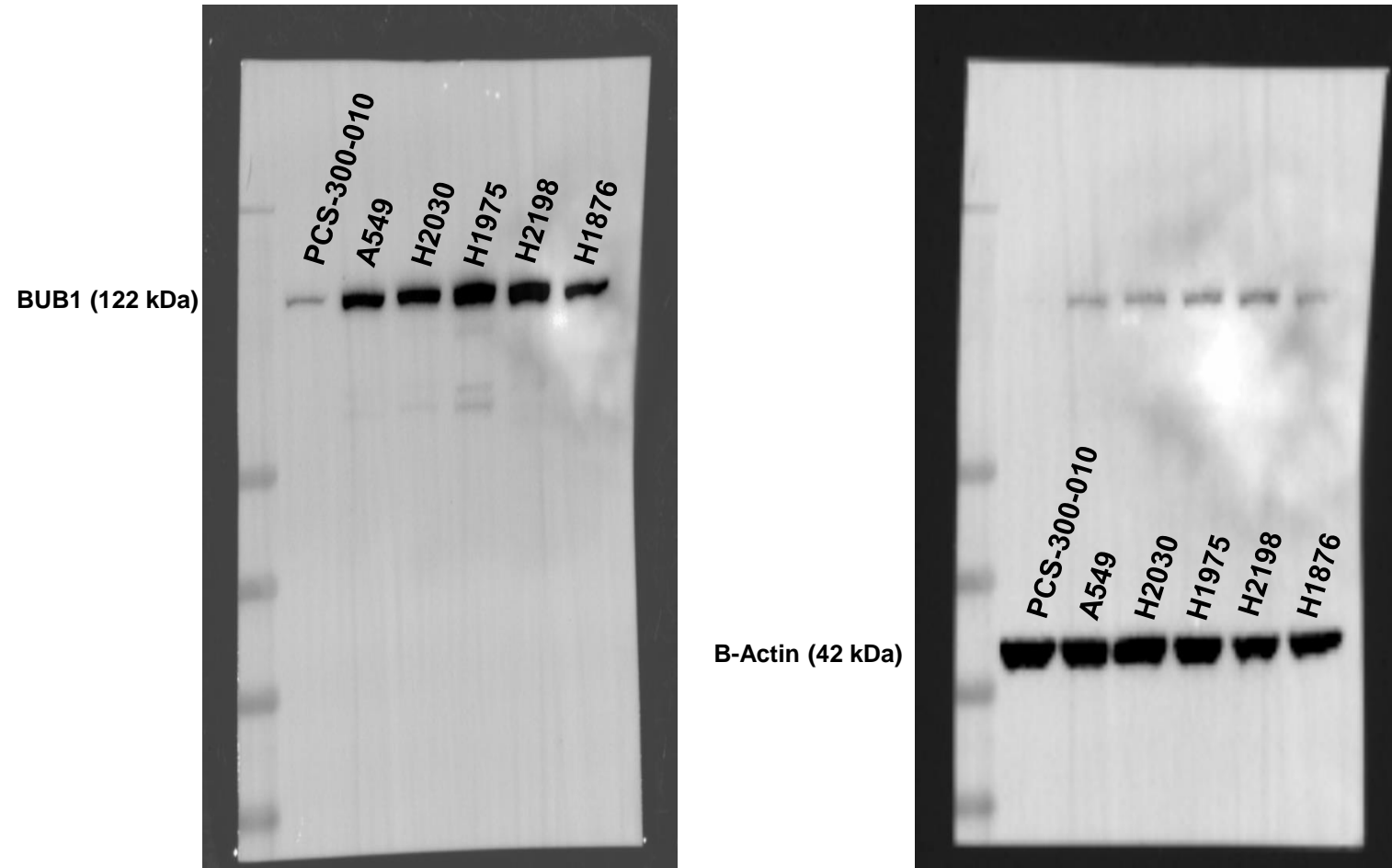

Supplement: Supplementary file 1 [file cancers-16-03291-s001.zip › cancers-3181850-File S1. The original Western blot figures.pdf]
